# Supplementary material for: Study on NGF and VEGF during the Equine Perinatal Period—Part 1: Healthy Foals Born from Normal Pregnancy and Parturition
Source: Vet Sci. 2022 Aug 23;9(9):451. doi: 10.3390/vetsci9090451 (PMC9504588; doi:10.3390/vetsci9090451)
Supplement: Supplementary file 1 [file vetsci-09-00451-s001.zip › vetsci-1843279-supplementary.pdf]

Table S1. Foals complete blood cell counts, serum biochemistry, electrolyte concentrations and rapid determinations at birth. Data are expressed as mean ±standard deviation (min-max). For normal values refer to [27–30].

Haematology

Haemoglobin g/dL	Haematocrit %	Erythrocytes 10^6/μL	Platelets 10^3/μL	Leucocytes 10^3/μL	Lymphocytes 10^3/μL	Monocytes cells/μL	Neutrophils 10^3/μL	Eosinophils cells/μL	Basophils cells/μL
16.0 ±0.8 (14.5-17.6)	48.3 ±2.1 (44.8-52.0)	11.2 ±0.6 (10.4-12.1)	197.8 ±39.8 (134-274)	8.3 ±1.1 (6.4-10.3)	1.4 ±0.4 (1.1-2.4)	218.6 ±71.6 (80-340)	6.6 ±1.0 (4.9-8.1)	15.7 ±12.2 (0-40)	82.9 ±153.4 (10-610)
Normal haematology values in one day old foals [27]:									
12.0-16.6	32-46	8.2-11.0	129-409	4.9-11.7	0.7-2.1	70-390	3.4-9.6	0-20	0-30

Serum biochemistry

Creatine kinase IU/L	Total bilirubin mg/dL	Triglycerides mg/dL	Total protein g/dL	Albumin g/dL	Albumin / Globulin	Blood urea nitrogen mg/dL	Creatinine mg/dL	Fibrinogen g/L	Serum amyloid A μg/dL
232 ±93 (102-370)	2.4 ±0.8 (1.6-4.6)	11.1 ±6.3 (2-24)	4.1 ±0.2 (3.8-4.7)	3.3 ±0.3 (2.8-3.8)	4.3 ±1.2 (2.9-7.0)	35.2 ±4.7 (28.0-42.4)	2.6 ±0.4 (1.9-3.5)	1.6 ±0.2 (1.4-2.0)	7.9 ±7.0 (1-23)
Normal serum biochemistry values in one day old foals [28,29]:									
40-909	1.3-4.5	30-193	4.3-8.1	2.5-3.6		9-40	1.2-4.3	1-4	0-37

Electrolyte concentrations

Phosphorus mg/dL	Calcium mg/dL	Sodium mg/dL	Potassium mg/dL	Chlorine mg/dL	Magnesium mg/dL
5.6 ±0.8 (4.1-6.9)	13.0 ±0.5 (12.3-14.2)	142.5 ±1.6 (140-145)	4.7 ±1.6 (4.0-5.7)	101.7 ±2.7 (96.9-104.9)	1.9 ±2.1 (1.7-2.1)
Normal electrolyte concentrations in one day old foals [28]:					
3.8-7.4	9.7-13.7	123-159	3.6-5.6	90-114	0.6-4.2

Rapid determinations

Jugular vein glucose mg/dL	Umbilical vein lactate mmol/L	Jugular vein lactate mmol/L
90.6 ±20.1 (51-127)	4.3 ±3.9 (0.5-15.1)	3.6 ±2.1 (0.5-8.5)
Normal determinations in foals at birth [28,30]:		
67-99	3.2-4.7	2.3-5.0

References

27. Harvey, J.W. Normal hematological values. In *Equine Clinical Neonatology*; Koterba, A.M., Drummond, W.H., Kosch, P.C., Eds.; Lea and Febiger: Philadelphia, 1990; pp. 561–570.
28. Bauer, J.E.; Harvey, J.W.; Asquith, R.L.; McNulty, P.K.; Kivipelto, J.A.N. Clinical chemistry reference values of foals during the first year of life. *Equine Vet. J.* **1984**, *16*, 361–363.
29. Stoneham, S.J.; Palmer, L.; Cash, R.; Rossdale, P.D. Measurement of serum amyloid A in the neonatal foal using a latex agglutination immunoturbidimetric assay: determination of the normal range, variation with age and response to disease. *Equine Vet. J.* **2001**, *33*, 599–603.
30. Pirrone, A.; Mariella, J.; Gentilini, F.; Castagnetti, C. Amniotic fluid and blood lactate concentrations in mares and foals in the early postpartum period. *Theriogenology* **2012**, *78*, 1182-1189.
